# GLP-1R/NPY2R regulate gene expression, ovarian and adrenal morphology in HFD mice

**DOI:** 10.1530/JOE-24-0189

**Published:** 2025-01-10

**Authors:** Dawood Khan, Ananyaa Sridhar, Charlotte R Moffett

**Affiliations:** Diabetes Research Group, School of Biomedical Sciences, Ulster University, Coleraine, Northern Ireland, UK

**Keywords:** high-fat diet, incretin, GLP-1R, NPYR, ovary, adrenal

## Abstract

Glucagon-like peptide-1 receptor (GLP-1R) and neuropeptide Y receptors (NPYRs) are expressed in reproductive tissues contributing to the regulation of gonadal function. This exploratory study examines the potential impact of their modulation by assessing the effects of exendin-4 (Ex-4) and peptide YY (PYY) (3–36) on endocrine ovaries and adrenals in high-fat diet (HFD) mice. Ex-4 and PYY(3–36) reduced blood glucose and energy intake, with no effects on body weight. While HFD did not impact the estrous cycle, Ex-4 increased metestrus frequency and decreased diestrus frequency resulting in 0% mice experiencing repeated diestrus or becoming acyclic. Luteinizing hormone levels were significantly higher in the Ex-4 and PYY(3–36) groups compared to the normal diet and HFD controls. In the adrenals, reduced capsule and zona glomerulosa thickness caused by HFD was reversed after peptide treatments. Within the ovaries, HFD increased the number of atretic follicles, an effect that disappeared after Ex-4 and PYY(3–36) treatments. Ex-4 also increased the number of corpora lutea owing to the prolonged metestrus phase. Gene expression analysis within the adrenals revealed the upregulation of *Insr* and the downregulation of *Prgtr* in HFD mice, while Ex-4 downregulated the expression of *Gipr*. The ovarian gene expression of *Gipr, Npy1r* and *Prgtr* was downregulated by Ex-4 treatment, while PYY(3–36) significantly downregulated the *Prgtr* expression compared to HFD mice. These data indicate that manipulating GLP-1R and NPY2R leads to changes in the reproductive physiology of mice. In addition, the observed alterations in the morphology and gene expression in the adrenals and ovaries imply a direct impact of these peptides on female reproductive function.

## Introduction

Obesity is a global epidemic, which continues to escalate across all populations. Lifestyle factors, including reduced physical activity and increased consumption of high-sugar and high-fat diets (HFDs), contribute to a positive energy balance (Romieu *et al.* 2017). Consequently, a surge in weight-related complications is evident, with well-established associations to type 2 diabetes, obstructive sleep apnea syndrome, cardiovascular ailments and liver diseases (Protasiewicz Timofticiuc *et al.* 2022). Infertility has emerged as a significant concern among the various complications associated with metabolic disorders, particularly in relation to polycystic ovary syndrome (PCOS) (Cena *et al.* 2020). A considerable proportion of women with PCOS (38–88%) are either overweight or obese (Barber *et al.* 2019). The syndrome manifests through anovulation, menstrual irregularities, insulin resistance and hyperandrogenism, affecting approximately 5–20% of women of reproductive age (Yao *et al.* 2021, Zhang *et al.* 2022). Our studies, along with others, have clearly demonstrated that HFD feeding in rodents leads to reproductive dysfunction, insulin resistance and irregular estrous cycles (Roberts *et al.* 2017, Khan *et al.* 2023). Obesity induced by HFD has been linked to menstrual or estrous cycle disruption, likely due to dysregulated endocrine mechanisms (Silvestris *et al.* 2018).

Despite systemic insulin resistance under HFD conditions, tissues of the hypothalamic–pituitary–ovarian axis remain insulin-sensitive (Brothers *et al.* 2010, Wu *et al.* 2012), suggesting that overstimulation of insulin signaling in the ovaries and hypothalamus may be a contributing factor. In addition, HFD has been shown to induce oxidative stress in the ovaries, affecting follicular development, survival and hormone production, which are critical for regulating folliculogenesis (Di Berardino *et al.* 2024). This oxidative damage negatively impacts fertility by impairing oocyte spindle formation and chromosome alignment, leading to abnormal meiotic events (Jia *et al.* 2018). Given the complex interplay between diet, lifestyle and ovarian health, a holistic approach is needed to address obesity-related reproductive dysfunction. Therefore, targeting key receptors of gut–reproductive axis that regulate energy and fertility may be the best path forward. Evidence of the expression of various gut hormone receptors in female reproductive tissues including glucagon-like peptide 1 (GLP-1), glucose-dependent insulinotropic polypeptide (GIP) and peptide YY (PYY) suggests an active role for these hormones in reproductive function and secretion (Ding *et al.* 2017, Urata *et al.* 2020, Khan *et al.* 2022). Recent studies have further demonstrated that these receptors are expressed in the ovaries and adrenals, with their expression levels being altered in conditions such as obesity and diabetes (Khan *et al.* 2023).

A novel direction in the management of obesity and diabetes has emerged through the utilization of GLP-1R agonists, which stimulate insulin release in a glucose-dependent manner. A recent study showed that the administration of GLP-1R agonist in mice with dehydroepiandrosterone-induced PCOS resulted in reduction of hyperinsulinemia and hyperandrogenemia (Zhang *et al.* 2023*a*,*b*). Furthermore, we previously highlighted the involvement of incretin hormones in female reproductive functions evidenced by disrupted estrous cycling and compromised reproductive outcomes in mice with global knockouts of *Gipr* and GLP-1R (Khan *et al.* 2022). Candidates including exenatide, liraglutide and semaglutide are common GLP-1R agonists in treatment related to metabolic disorders (Wang *et al.* 2023). Exendin-4, a naturally occurring 39-amino acid reptilian peptide, is a hypoglycemic drug, which shares a sequence homology of 53% with GLP-1R (Yin *et al.* 2005). Extensive research has shown that Ex-4 significantly reduces food intake when administered both centrally and peripherally (Hayes *et al.* 2011, Yang *et al.* 2014). Moreover, Ex-4’s anorexigenic effects are mediated through sensory afferent pathways involving GLP-1Rs and reward-related mechanisms, which are distinct from the satiety-driven pathways utilized by hormones such as PYY and CCK (Talsania *et al.* 2005, Lopez *et al.* 2023). Recent study by Simpson and coworkers shows that GLP-1R modulation by Ex-4 stimulates luteinizing hormone (LH) secretion in sheep in follicular and luteal phase (Simpson *et al.* 2023). In harmony, studies have indicated that GLP-1R agonists exhibit the potential to ameliorate menstrual irregularities in patients with PCOS (Han *et al.* 2019).

PYY is a 36-amino acid peptide that is in circulation in two major forms PYY(1–36) and PYY(3–36) (Khan *et al.* 2016). Neuropeptide Y (NPY) and PYY share five NPY G-protein-coupled receptors (Y1, Y2, Y4, Y5 and Y6) with NPY2R specific to PYY(3–36), while PYY(1–36) binds to all NPY receptors (Ruscica *et al.* 2007, Khan *et al.* 2016). Both NPY and PYY(3–36) control eating behavior, where hypothalamic NPY centrally regulates sexual behavior and reproductive functions (Chen *et al.* 2023). Furthermore, PYY administration delayed the estradiol-induced LH surge in ovariectomized ewes (Clarke *et al.* 2005). However, the physiological relevance of the GLP-1R and NPYR to reproductive axis is not well known. In the past decade, research in the field of gastric bypass surgery has spotlighted the role of gut hormones as direct contributors to the positive impacts on female fertility. Altered postoperative release of gut peptides and their cellular adaptations in pancreas and intestines are recognized as contributing factors to the improvement of type 2 diabetes following surgery (Sridhar *et al.* 2022, Khan *et al.* 2023). Furthermore, a recent clinical trial has shown that bariatric surgery is more effective than medical treatment in inducing spontaneous ovulation in women with PCOS, obesity and oligomenorrhea or amenorrhea (Samarasinghe *et al.* 2024).

Despite the abundance of published studies exploring the roles of obesity and PCOS, there remains a notable gap concerning the morphological transformations in ovaries/adrenals, modifications to reproductive hormones and alterations in gut hormone receptors. Therefore, the present study investigated the potential therapeutic role of extrinsic modulators of NPY2R and GLP-1R in estrous cycling, changes in circulating reproductive hormones and their concurrent effects on ovarian and adrenal morphology. A vital aspect of this study involves an exploration of whether observed changes in adrenal and ovarian morphology correlate with the abnormal reproductive function. The presented data support the proposition that high-fat feeding disrupts female reproductive function and modulation of key incretin receptors may be used as therapeutic candidates for energy-related female infertility.

## Materials and methods

### Animals

**Figure 1:**
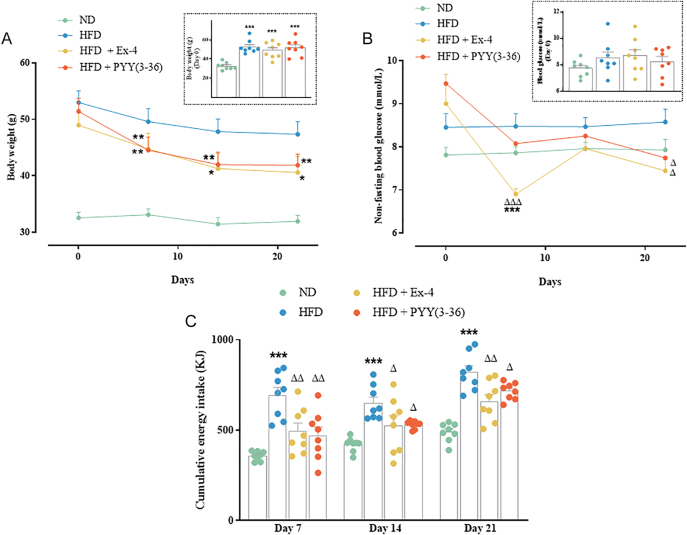
Effect of Ex-4 and PYY(3–36) on metabolic parameters of high-fat-fed female mice. (A) Body weight (g); the inset graph illustrates body weight at day 0, (B) non-fasting blood glucose (mmol/L), the inset graph illustrates blood glucose at day 0, and (C) cumulative energy intake (KJ). Values are mean ± SEM (*n* = 8). *P < 0.05, **P < 0.01 and ***P < 0.001 compared to ND control mice; ^Δ^*P* < 0.05, ^ΔΔ^*P* < 0.01 and ^ΔΔΔ^*P* < 0.001 compared to HFD mice.

Female NIH Swiss mice (4–6 weeks old, Envigo, UK) were housed individually in an air-conditioned room at 22 ± 2°C with 12 h light and darkness cycle and access to drinking water and standard rodent diet referred to here as normal diet (ND) (10% fat, 30% protein and 60% carbohydrate; 12.99 kJ/g, Trouw Nutrition, UK) was provided *ad libitum*. At 9 weeks of age, mice were fed a HFD (45% fat, 35% carbohydrate and 20% protein; 26.15 kJ/g, Special Diet Services, UK) for 14 weeks, which resulted in increased body weight (Fig. 1). Following this, three groups of HFD mice (*n* = 8) were administered i.p. injections of either saline vehicle (0.9% NaCl), Ex-4 or PYY(3–36) (25 nmol/kg body weight, Synpeptide Co. Ltd., China) twice daily for 21 days with a separate saline-treated ND group of mice employed as controls. The metabolic parameters measured were body weight, non-fasting blood glucose and energy intake. At termination, animals were sacrificed by lethal inhalation of CO_2_ followed by cervical dislocation. All experiments were conducted under the UK Animals (Scientific Procedures) Act 1986, the EU Directive 2010/63EU and the UK Home Office Animal Project Licence Number PPL2902 and approved by the Ulster University’s Animal Welfare and Ethical Review Body (AWERB).

### Assessment of stages of estrous cycle

Assessments of estrous cycle stages were carried out as described previously using wet vaginal smears taken every day from day 4 until day 21 from conscious mice between 11:00 h and 12:00 h (Khan *et al.* 2023). Samples were taken at approximately the same time of the day over the course of the collection period to reduce variability. During assessment, the tail was elevated to visualize the vagina. For obtaining wet smear, the vaginal cells were flushed by gently introducing a little amount (100 μL) of distilled water or saline using a pipette. A new pipette was used for each animal. The liquid was slowly released into the vagina and drawn back into the tip; this was repeated about 4–5 times with the same sterile tip. Care was taken not to insert the tip too deep to avoid cervical stimulation as excessive stimulation can induce pseudopregnancy. The final fluid containing about 10 μL solution was collected in an Eppendorf. Later, the collected sample of vaginal epithelial cells was placed on a clean glass slide in a thin layer and observed under light microscope (Olympus IX51) with 10× objective lens.

### Tissue processing

Adrenal and ovarian tissues were extracted from mice and fixed for 48 h in paraformaldehyde (4% w/v) to preserve cellular architecture by cross-linking proteins. Tissues were then processed in an automated tissue processor as described (Leica TP1020, Leica Microsystems, Germany), which involved dehydrating tissues in 70–100% ethanol, followed by xylene immersion to remove wax before paraffin embedding (Khan *et al.* 2019). Tissue blocks were sectioned (6 μm) using a Shandon Finesse 325 microtome (Thermo Scientific, UK) and picked for staining at intervals of 10 sections, placed on poly-L-lysine-coated slides.

### Hematoxylin and eosin staining

Slides containing tissues were dewaxed in xylene and then rehydrated using a series of ethanol solutions. The sections were exposed to hematoxylin solution for 5 min and rinsed with tap water, acid alcohol (0.25% HCl, 50% methanol) and again in tap water before staining with eosin for 5 min. Following rinsing with distilled water, sections were dehydrated using ethanol, dipped in histo-clear II for 2 min and mounted using a DePeX mounting medium. The slides were then scanned using NanoZoomer digital pathology software (NDP.serve 3.3.30) at University College London. The ruler function was used to measure the thickness of the adrenal capsule and the zona glomerulosa with six measurements each, and the results were averaged per animal.

### Biochemical analysis

Non-fasting plasma glucose was directly measured from the cut tip on the tail vein of conscious mice between 13:00 and 14:00 h using a hand-held Ascensia Contour blood glucose meter (Bayer Healthcare, UK). At the end of treatment period, plasma was collected and immediately centrifuged at 13,000 *g* and stored at −70°C. Reproductive hormones were determined by the Ligand Assay and Analysis Core (Center for Research in Reproduction, University of Virginia, USA).

### Real-time reverse transcription PCR

mRNA was extracted from the snap-frozen tissues (Khan *et al.* 2017) using a RNeasy Mini Kit following the manufacturer's instructions (Qiagen, UK). mRNA (150 ng) was reverse transcribed to cDNA using SuperScript II Reverse Transcriptase kit (Invitrogen, UK). qPCR was performed on a LightCycler 480 System (Roche, UK) using designed primers (Thermo Fisher Scientific). The reaction mixture for real-time PCR consisted of QuantiFast SYBR Green master mix (Qiagen, UK), primers (forward and reverse), cDNA and RNase-free water. Amplification conditions were set at 95°C for initial and final denaturation, 58°C for primer annealing and 72°C for extension for 40 cycles, followed by a melting curve analysis, with temperature range set at 60–90°C. To validate primers, an equalized pool of cDNA from each group was diluted 1:20 and initially tested using a thermal gradient to determine the optimal annealing temperature (58°C), average level of expression and unique product for each target from melt curve analysis. The quantitative cycle (Cq) value at 58°C was used to establish the standard curve dilution factor for each target. A no-template control was used to test for contamination of buffers and solutions. Data were analyzed using ΔΔCt method and normalized to GAPDH expression. Custom primers used in the study (Table 1) were obtained from Invitrogen, UK.

**Table 1:** List of mouse primers used.

Gene symbol	Alias/common name	Primer sequence (5′-nt-3′)
*Gapdh*	Glyceraldehyde-3-phosphate dehydrogenase	F-GGACCTCATGGCCTACATGG R-TAGGGCCTCTCTTGCTCAGT
*Glp1r*	Glucagon-like peptide 1 receptor	F-GGGCCAGTAGTGTGCTACAA R-CTTCACACTCCGACAGGTCC
*Gipr*	Glucose-dependent insulinotropic peptide receptor	F-TCACCTTTCAAGGATGCCCC R-GCCCCTCAGAGTCTGTCTCC
*Npy2r*	Neuropeptide Y2 receptor	F-GTAGGTGCAGAGGCAGATGAG R-CCAGAGCAATGACTCTAGGAGTAG
*11βHsd*	11-Beta dehydrogenase 1	F-CTGCCTGGGAGGTTGTAGAAA R-ATCAAACAGGGACCTGGCTC
*Insr*	Insulin receptor	F-ATGGTGCCGAGGACAGTAGG R-GAGTGTGGTGGCTGTCACAT
*Npy1r*	Neuropeptide Y1 receptor	F-TCCCTCCAGTGACACTCGTC R-ACAGAAAGAGTTTGCATCTCACT
*Ghsr*	Ghrelin receptor	F-CCGATAGAGTGACAGGCTTC R-TCCTAGGCGCGGAAGAGT
*Gcgr*	Glucagon receptor	F-CCGCCTAGTGTTCAAGAGGT R-AACTGACATTGGGAGGCGTT
*Amh*	Anti-Mullerian hormone	F-TCGGGCCTCATCTTAACCCT R-CGTGAAACAGCGGGAATCAG
*Esr1*	Estrogen receptor 1	F-CGCTCTGCCTTGATCACACA R-GCGAGTTACAGACTGGCTCC
*Npy5r*	Neuropeptide Y5 receptor	F-TCTCAAGCAGAAGCGACCG R-CTAGAGTCCTGCTCGGGATG
*Prgtr*	Progesterone receptor	F- CATGGTCCTTGGAGGTCGTA R- AGCAACACCGTCAAGGGTTC

### Statistical analysis

GraphPad Prism (version 8.0) software was used to perform statistical analysis. Values are expressed as mean ± S.E.M. Comparative analyses between groups were carried out using two-way or one-way ANOVA with Bonferroni post hoc test. There were no inclusion and exclusion criteria applied. Data of the groups were considered to be significant if *P* < 0.05.

## Results

### Effect of Ex-4 and PYY(3–36) on body weight, blood glucose and energy intake

As expected, the HFD group exhibited a significant (*P* < 0.001) increase in body weight throughout the study compared to the ND group (Fig. 1A). The elevated body weight persisted in the Ex-4 and PYY(3–36) groups, mirroring results observed in the HFD group (Fig. 1A). Non-fasting blood glucose decreased significantly (*P* < 0.001) on day 7 in the Ex-4 group compared to both ND and HFD controls (Fig. 1B). Following the administration of Ex-4 and PYY(3–36) for 21 days, there was a significant (*P* < 0.05) decrease in blood glucose compared to HFD mice (Fig. 1B). Ex-4 and PYY(3–36) consistently and significantly (*P* < 0.05 to *P* < 0.01) decreased energy intake over the 21-day period compared to HFD mice (Fig. 1C).

### Effect of Ex-4 and PYY(3–36) on estrous cycle

**Figure 2:**
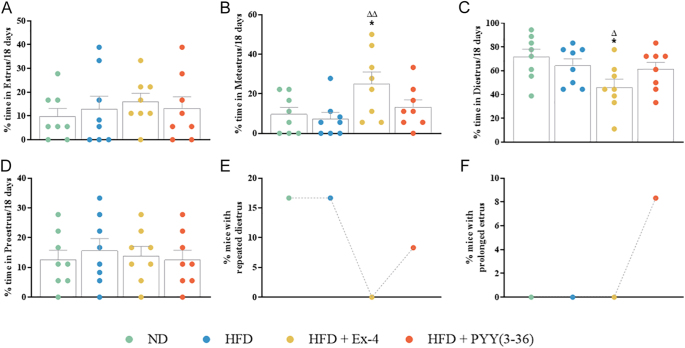
Effect of Ex-4 and PYY(3–36) on the estrous cycle of high-fat-fed female mice. (A) % time spent in estrus, (B) % time spent in metestrus, (C) % time spent in diestrus, (D) % time spent in proestrus, (E) % mice with repeated diestrus and (F) % mice with prolonged estrus. Values are mean ± SEM (*n* = 12). **P < 0.01 compared to ND control mice; ^Δ^*P* < 0.05 and ^ΔΔ^*P* < 0.01 compared to HFD mice.

Analysis of different stages of the estrous cycle revealed no significant alterations in the frequency of estrus, metestrus, diestrus and proestrus after HFD and PYY(3–36) compared to ND mice (Fig. 2A, B, C, D). However, Ex-4 significantly (*P* < 0.01) increased the frequency of metestrus compared to both ND and HFD groups (Fig. 2B). Correspondingly, Ex-4 significantly (*P* < 0.05 and *P* < 0.01) reduced the frequency of diestrus compared to ND and HFD mice (Fig. 2C). This reflected as 0% mice in the Ex-4 group exhibiting repeated diestrus as opposed to 16% in the ND and HFD and 8% in the PYY(3–36) group mice (Fig. 2E). A prolonged estrus stage was observed in 8% mice after PYY(3–36) treatment (Fig. 2F).

### Effect of Ex-4 and PYY(3–36) on circulating reproductive hormones

**Figure 3:**
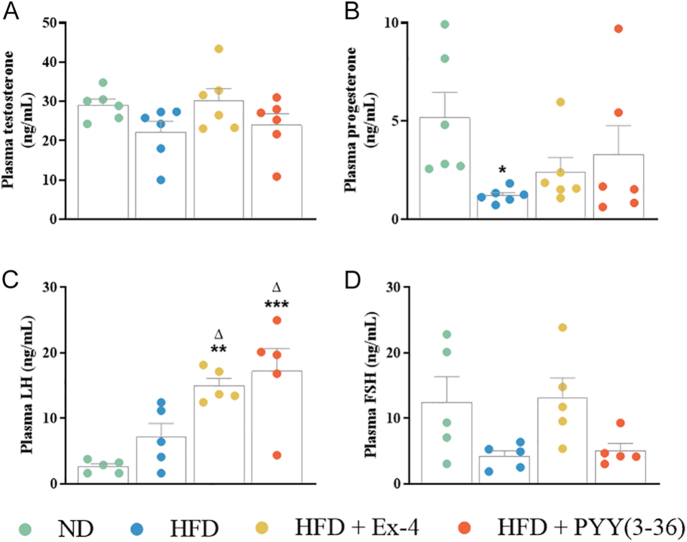
Effect of Ex-4 and PYY(3–36) on hormone measurement in the plasma of high-fat-fed female mice. (A) Testosterone, (B) progesterone, (C) LH and (D) FSH. Values are mean ± SEM (*n* = 5–6). *P < 0.05, **P < 0.01 and ***P < 0.001 compared to ND control mice; ^Δ^*P* < 0.05 compared to HFD mice.

Plasma testosterone levels remained unchanged after HFD and peptide treatments compared to ND mice (Fig. 3A). However, there was a significant (*P* < 0.05) decline in plasma progesterone levels in the HFD group compared to the ND group (Fig. 3B). Progesterone levels were not altered after peptide treatments when compared to HFD mice. Contrastingly, plasma LH significantly (*P* < 0.05 to *P* < 0.001) increased after Ex-4 and PYY(3–36) compared to ND and HFD mice (Fig. 3C). There was no change in plasma follicle-stimulating hormone (FSH) between the ND, HFD and peptide-treated groups (Fig. 3D).

### Effect of Ex-4 and PYY(3–36) on adrenal and ovarian morphology

**Figure 4:**
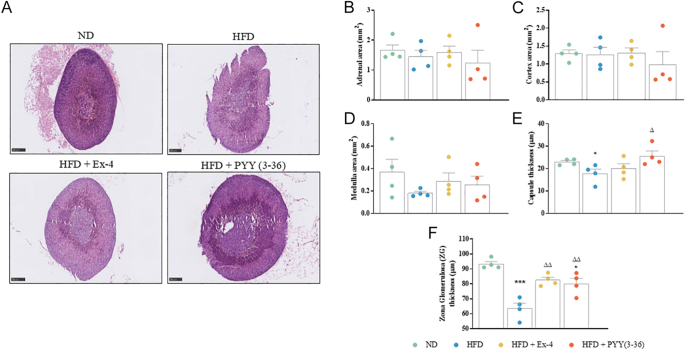
Effect of Ex-4 and PYY(3–36) on adrenal morphology of high-fat-fed female mice. (A) Representative images of adrenal glands stained for H&E, (B) adrenal area (mm^2^), (C) cortex area (mm^2^), (D) medulla area (mm^2^), (E) capsule thickness (μm) and (F) zona glomerulosa thickness (μm). Representative images were taken at 3–5× magnifications with appropriate scale bars included at 250 µm. Values are mean ± SEM (*n* = 4). **P < 0.01 and ***P < 0.001 compared to ND control mice; ^Δ^*P* < 0.05 and ^ΔΔ^*P* < 0.01 compared to HFD mice.

**Figure 5:**
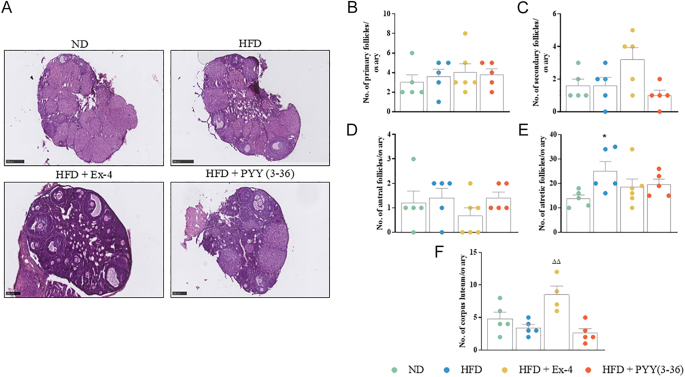
Effect of Ex-4 and PYY(3–36) on ovarian morphology in high-fat-fed female mice. (A) Representative images of ovaries stained for H&E, (B) number of primary follicles, (C) number of secondary follicles, (D) number of antral follicles, (E) number of atretic follicles and (F) number of corpus luteum. Representative images were taken at 3–5× magnifications with appropriate scale bars included at 250–500 μm. Values are mean ± SEM (*n* = 4–6). *P < 0.05 compared to ND control mice; ^ΔΔ^*P* < 0.01 compared to HFD mice.

Representative images of adrenals and ovaries stained for H&E are shown in Figs 4A and 5A, respectively. In the adrenal gland, HFD and peptide treatments did not cause any significant alterations in adrenal, cortex and medulla area (Fig. 4B, C, D). Capsule thickness decreased significantly (*P* < 0.05) in the HFD group compared to the ND group (Fig. 4E). PYY(3–36) treatment significantly (*P* < 0.05) increased capsule thickness to ND levels (Fig. 4E). Correspondingly, ZG thickness decreased significantly (*P* < 0.001) after HFD (Fig. 4F). Both Ex-4 and PYY(3–36) significantly (*P* < 0.01) increased ZG thickness in the adrenal gland (Fig. 4F). In the ovary, there was no significant difference in the number of primary, secondary or antral follicles in the HFD, Ex-4 and PYY(3–36) groups when compared to ND controls (Fig. 5B, C, D). The number of atretic follicles increased significantly (*P* < 0.05) in the HFD group compared to the ND group (Fig. 5E). The corpus luteum count increased significantly (*P* < 0.01) in the Ex-4 group compared to the HFD group (Fig. 5F).

### Effect of EE2 on gene expression in the adrenals and ovaries

**Figure 6:**
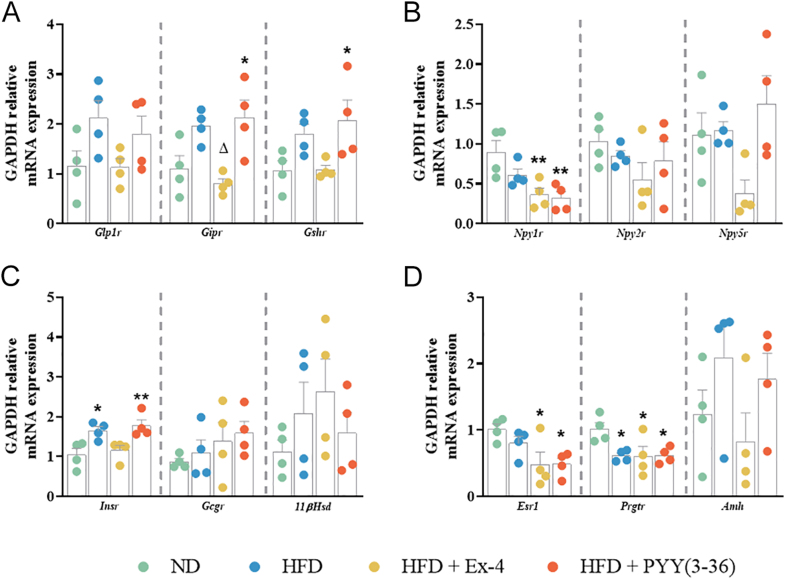
Effect of Ex-4 and PYY(3–36) on relative mRNA expression in the adrenals of high-fat-fed female mice. GAPDH relative mRNA expression of (A) *Glp1r/Gipr/Gshr*, (B) *Npy1r/Npy2r/Npy5r*, (C) *Insr/Gcgr/11βHsd* and (D) *Esr1/Prgtr/Amh*. Values are mean ± SEM (*n* = 4). *P < 0.05 and **P < 0.01 compared to ND control mice; ^Δ^*P* < 0.05 compared to HFD mice.

**Figure 7:**
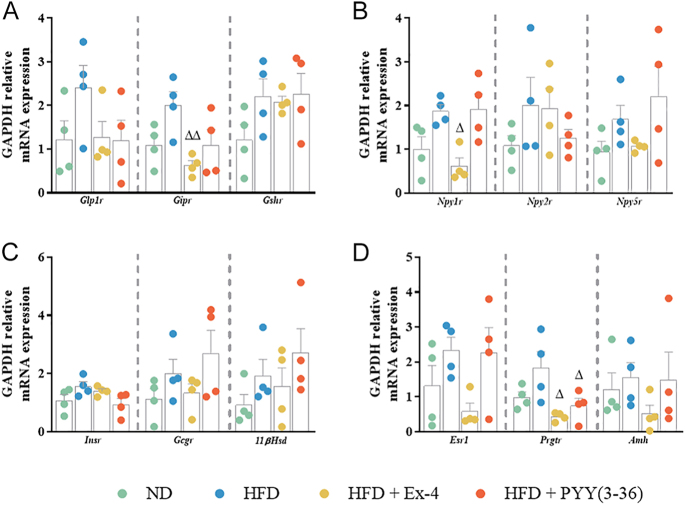
Effect of Ex-4 and PYY(3–36) on relative mRNA expression in the ovaries of high-fat-fed female mice. *Gapdh* relative mRNA expression of (A) *Glp1r/Gipr/Gshr*, (B) *Npy1r/Npy2r/Npy5r*, (C) *Insr/Gcgr/11βHsd* and (D) *Esr1/Prgtr/Amh*. Values are mean ± SEM (*n* = 4). *P < 0.05 and **P < 0.01 compared to ND control mice; ^Δ^*P* < 0.05 compared to HFD mice.

Adrenal expression of *Glp1r, Gipr, Gshr, Npy1r, Npy2r, Npy5r, Gcgr, 11βHsd, Esr1 and Amh* remained unchanged in HFD mice (Fig. 6A, B, C, D). However, HFD significantly (*P* < 0.05) upregulated *Insr* and downregulated *Prgtr* expression in the adrenals (Fig. 6C and D). Ex-4 significantly downregulated *Gipr* expression in adrenals of HFD mice (Fig. 6A). Compared to the ND controls, Ex-4 and PYY(3–36) significantly (*P* < 0.05 to *P* < 0.01) downregulated *Npy1r* and *Esr1* expression (Fig. 6B and D). PYY(3–36) maintained a significantly (*P* < 0.01) higher expression of *Insr* as observed in HFD mice (Fig. 6C). In the ovaries, HFD did not alter gene expression, while Ex-4 significantly (*P* < 0.05) downregulated *Gipr, Npy1r* and *Prgtr* compared to HFD mice (Fig. 7A, B, C, D). Similarly, PYY(3–36) significantly (*P* < 0.05) downregulated *Prgtr* expression in the ovaries (Fig. 7D).

## Discussion

The idea that gut hormones have an important role to play in the regulation of female reproductive function is gaining more acceptance (Han *et al.* 2019, Moffett & Naughton 2020, Khan *et al.* 2022, Khan *et al.* 2023). For example, the administration of liraglutide leads to substantial reductions in weight and testosterone levels, yielding mixed outcomes related to menstrual patterns (Cena *et al.* 2020). Moreover, there is emerging evidence of adaptive roles of GLP-1R, GIP, ghrelin and PYY among others within the gut, especially post-gastric bypass surgery that has proven beneficial in fertility outcomes (Khan & Moffett 2020, Moffett & Naughton 2020). We have previously confirmed the expression of GLP-1R and GIPR in female mice reproductive tissues and how global incretin receptor deletion impacted female fertility and pregnancy outcomes (Khan *et al.* 2022). While not many studies have been conducted on the relationship between NPYR and reproduction, few suggest that NPY serves as a physiological stimulus, promoting the release of GnRH before ovulation (Manfredi-Lozano *et al.* 2018). Altogether, this would suggest a role of GLP-1R and NPYR in the regulation of female reproductive function.

As expected, feeding female NIH Swiss mice with HFD for 14 weeks resulted in increased body weight with minimal changes in blood glucose levels. In this regard, direct positive effects of Ex-4 and PYY(3–36) improving glycemia in female obese mice were observed. Previous studies have demonstrated that chronic administration of Ex-4 and PYY can improve glucose responsiveness (Arakawa *et al.* 2009, Guida & Ramracheya 2020, Sridhar *et al.* 2023), a parameter not assessed in our study. Moreover, most of these studies were performed on male rodent models and further reporting is required on female counterparts for gender-specific differences. Consistent with other research indicating the anorexigenic effects of Ex-4 and PYY(3–36), we noted substantial reduction in feeding in female mice, following the administration regime (Arakawa *et al.* 2009, Sridhar *et al.* 2023). This finding is particularly relevant considering that increased risk of obesity has been linked to a higher incidence of reproductive disorders, including anovulation, menstrual irregularities, infertility, challenges in assisted reproduction, miscarriage and adverse pregnancy outcomes (Dag & Dilbaz 2015). Thus, the impact of these peptides on feeding behavior may have broader implications for addressing obesity-related reproductive issues.

Estrous cycle monitoring revealed no changes in the time spent in each phase of the estrous cycle after HFD. This was surprising as previously, studies have shown disrupted estrous cycling in HFD rodents (Hohos *et al.* 2018, Khan *et al.* 2023). The duration/composition of HFD and species/strain variations could affect the extent of impact on estrous cycle (Barkley & Bradford 1981). Interestingly, Ex-4 prolonged metestrus while shortening time in diestrus that comprises the luteal phase of the cycle. While the precise mechanisms underlying this phenomenon remains unclear, it is possible that the phases in question are linked to corpus luteum function. In the ovary, prolonged metestrus was accompanied by an increased number of corpora lutea that form during metestrus. During the luteal phase, progesterone levels typically rise (Ajayi & Akhigbe 2020), but we observed no changes in plasma progesterone levels following Ex-4 administration. Consequently, the optimal function of corpus luteum during the diestrus period may be compromised, potentially due to the shortened duration of the diestrus phase. We did not observe changes in estrous cycle stages following PYY(3–36), possibly due to the minimal effects of PYY, as a previous study reported that PYY concentrations do not significantly vary between cycle stages in rats (Johnson *et al.* 2017). However, intraperitoneal administration of PYY(3–36) did result in a prolonged estrus phase lasting 5 days. This extension of the follicular phase may lead to delayed ovulation. Hence, the involvement of other NPYRs in reproductive cycle cannot be ruled out. The effect of other NPYR modulators on specific phases of the estrous cycle requires further investigation and may unravel the role of NPY family of peptides in fertility.

HFD reduced plasma progesterone causing loss of its inhibitory effect on estradiol-mediated LH surge. Increased plasma LH may be attributed to reduced progesterone, which is shown to block LH surge through its receptor in the anteroventral periventricular nucleus of the hypothalamus (Liu *et al.* 2020). Previous studies have shown varying effects of HFD on circulating hormone levels and seem to be fluctuating relative to estrous cycle phases (Negrón & Radovick 2020). Interestingly, GLP-1R and NPY2R activation with Ex-4 and PYY(3–36) increased circulatory LH. GLP-1R agonists have been shown to activate kisspeptin action in the arcuate nucleus in brain slices (Heppner *et al.* 2017) and to increase kisspeptin mRNA expression *in vitro* (Oride *et al.* 2017). Kisspeptin is a potent stimulator of GnRH (Gottsch *et al.* 2004), which could explain the observed increase in plasma LH levels following Ex-4 administration. In addition, PYY(3–36) has been found to directly stimulate LH secretion from the isolated pituitary of prepubertal female rats (Fernandez-Fernandez *et al.* 2005). Our data suggest that this effect may persist after puberty.

Morphological examination of the ovaries did not show any major differences in primary, secondary or antral follicle counts. Consistent with previous findings, our study revealed that obesity resulted in a significant elevation of atretic follicles in mice (Hilal *et al.* 2020). However, this was countered by both Ex-4 and PYY(3–36). The mechanisms by which PYY(3–36) influences follicular apoptosis remain unclear; however, a previous study has shown that GLP-1R agonism, *via* liraglutide, can reduce apoptosis in mouse granulosa cells through phosphorylation modification of FoxO1 expression (Sun *et al.* 2020). Thus, the modulation of GLP-1R and NPY2R leading to decreased numbers of atretic follicles might be associated with the inhibition of follicular apoptosis.

Besides the ovaries, the adrenal glands also play an important role in steroidogenesis. While the existence of zona reticularis and androgen secretion from the mouse adrenal gland has been a topic of debate (Dumontet & Martinez 2021), initial studies indicate that the weight of the rat adrenal gland varies across different phases of the estrus cycle, being higher during estrus compared to during diestrus (Andersen & Kennedy 1932). Our examination of the adrenal gland morphology revealed no alterations in the total adrenal area, cortex area or medulla area of HFD mice. However, this finding contrasts with a prior study that reported cortical hyperplasia in male mice, suggesting that gender-specific differences may underlie these disparate results (Swierczynska *et al.* 2015). The adrenal capsule serves as a pivotal signaling center, essential for the replenishment of damaged cells and maintaining zonation (Vidal *et al.* 2016). The HFD could potentially disrupt this process, as evidenced by its reduction in capsule and zona glomerulosa thickness, which were restored to normal levels by Ex-4 and PYY(3–36). Notably, PYY(3–36) further increased capsule thickness, suggesting a direct influence of NPY2R on adrenal function. Previous studies have demonstrated the impact of PYY on adrenals both *in vitro* and *in vivo* (Neri *et al.* 1991), along with the involvement of *Npy1r* (Renshaw *et al.* 2000). Therefore, it is evident that NPYRs directly affect adrenal morphology, potentially influencing its functions under stress conditions such as diet-induced obesity. The reduction in zona glomerulosa thickness following HFD may represent a compensatory mechanism, as obesity is associated with aldosterone excess and direct associations between aldosterone deficiency and diet-induced obesity have been reported earlier (Luo *et al.* 2013, Liao *et al.* 2018). However, other studies using HFD male mice did not observe any effects on the zona glomerulosa (Swierczynska *et al.* 2015, Navarrete *et al.* 2018). It is well-established that fluctuations in LH and FSH influence ovarian morphology (Egbert *et al.* 2019) and adrenal steroidogenesis (Kero *et al.* 2000); however, under stress conditions such as diet-induced obesity, local effects of gut hormones may also contribute to the regulation of these processes. It is important to note that the estrous cycle stage influences plasma hormone concentrations and ovarian/adrenal morphology. Our primary objective was to observe patterns that are more applicable across different phases of the estrous cycle, rather than limiting the findings to a specific time point. Since this variable was not strictly controlled for, it represents a potential limitation. Determining the terminal estrous phase at the time of sample collection would have allowed for more precise phase-related correlations, helping to rule out this factor as a source of variability. Another limitation of this study is the relatively small sample size, and future research with larger cohorts will be necessary to expand upon these findings.

To further probe the link between gut–reproductive axis, we conducted comprehensive mRNA quantification studies, focusing on key gut hormone receptors present in both adrenals and ovaries. Adrenal *Insr* expression increased with HFD but decreased to ND levels with Ex-4 treatment. This supports the idea that insulin signaling activation can play a direct role, at least partially, in regulating steroidogenesis in the adrenal gland (Kinyua *et al.* 2018, Werdermann *et al.* 2021). Furthermore, our findings confirm the direct impact of GLP-1R on adrenal steroidogenesis. HFD downregulated the expression of *Prgtr* in the adrenal glands. Previous rodent studies have confirmed that progesterone is secreted by the adrenals (Fajer *et al.* 1971) and that *Prgtr* is expressed in the adrenal capsule of mice (Uotinen *et al.* 1999). Although the specific function of *Prgtr* in the adrenals of rodents remains unclear, its downregulation in HFD mice appears to correlate with a decrease in circulating progesterone. In relation to the expression of *Gipr*, no alteration was observed with HFD, whereas treatment with Ex-4 led to a significant reduction in its expression. This phenomenon may be attributed to GIP’s role in enhancing glucocorticoid secretion from the adrenal glands, and limiting this pathway can potentially amplify the anorexigenic properties of Ex-4 (Lee *et al.* 2016). In addition, in humans, exenatide has been shown to prevent glucocorticoid-induced glucose intolerance and islet cell dysfunction (Van Raalte *et al.* 2011). Increased cortisol levels resulting from ACTH stimulation in the adrenal glands have been observed in women with PCOS (Fujii *et al.* 2014). PYY(3–36) administration resulted in an increase in the expression of *Gipr* and *Gshr* within the adrenal glands. This observed alteration, coupled with the concurrent reduction in *Npy1r* and *Esr1* expression induced by both peptides, warrants deeper investigation. The changes caused by PYY(3–36) suggest potential crosstalk between *Npy2r* activation and *Npy1r*.

mRNA quantification in the ovaries revealed that HFD had no significant effects; however, treatment with Ex-4 resulted in a marked downregulation of *Gipr* and *Npy1r* expression. While recent studies have underscored the beneficial effects of GLP-1R agonists in the context of PCOS (Nylander *et al.* 2017, Zhang *et al.* 2023*a*,*b*, Zhou *et al.* 2023), their impact on the ovaries in diet-induced obesity remains unclear. Although we did not observe an upregulation of *Glp-1r* in the ovary, the reduction in *Gipr* suggests that Ex-4 might indirectly regulate steroidogenesis. *Gipr* is known to modulate ovarian steroidogenesis through the upregulation of BMP receptor signaling (Nishiyama *et al.* 2018). In addition, Ex-4 induced a decrease in *Npy1r* expression, indicating that Ex-4 may control the direct effects of NPY on ovarian cell proliferation and apoptosis. The specific actions of NPY remain ambiguous as some studies report that NPY inhibits proliferation and promotes apoptosis via the p53 protein (Sirotkin *et al.* 2015), while others suggest a stage-dependent action (Urata *et al.* 2020) or no effect under normal conditions, although hyperandrogenism may alter this response (Urata *et al.* 2023). Another notable finding was the downregulation of *Prgtr* by both Ex-4 and PYY(3–36). *Prgtr* expression varies among different cell types in the ovary across different stages of the estrous cycle (Gava *et al.* 2004). Previous research has shown that *Glp-1* treatment significantly suppresses progesterone synthesis in the presence of FSH in rat granulosa cells (Nishiyama *et al.* 2018). Further investigation into how local activation of *Glp1r* and *Npy2r* influences various ovarian cell types throughout the estrous cycle under conditions of cellular stress will enhance our understanding of the mechanisms underlying energy-related reproductive dysfunction. The observed effects could be due to the peptide treatment or reduced food intake, making it challenging to definitively attribute the outcomes solely to peptide administration. Previous studies suggest that the effects of peripheral administration of Ex-4 and PYY(3–36) are often primarily associated with reduced food intake (Yang *et al.* 2014, Jones *et al.* 2019). However, the presence of their receptors in the ovaries and adrenals implies that there may also be direct effects independent of changes in food intake. Further investigation using a pair-fed analysis would be valuable to elucidate these potential direct effects. Our data indicate that the activation of these receptors can modulate the expression of other critical receptors within the ovaries highlighting the significance of the gut–reproductive axis in the regulation of female metabolic processes.

## Conclusion

This exploratory study is a new chapter in bridging critical knowledge gaps in our understanding of the gut–reproductive connection. Our investigation started with the global deletion of incretin receptors (Khan *et al.* 2022), moving toward unraveling the detrimental effects of HFD on female reproductive outcomes (Khan *et al.* 2023). The present investigation propels the narrative forward, shedding light on the extrinsic activation of GLP*-*1 and NPY receptors. In conclusion, Ex-4 may help protect against follicular atresia and restore adrenal morphology, potentially by modulating gene expression in the ovaries and adrenals in HFD-fed rodents. In addition, changes in circulatory LH levels following Ex-4 and PYY(3–36) administration, possibly linked to altered estrous cycling, underscore the significance of the gut–reproductive axis in females. While these findings must be replicated in additional cohorts, it opens the possibility of further investigations into whether knocking out GLP-1 receptor specifically in the ovaries could prevent alterations in gene expression and hormone release. Taken together, these data suggest that direct activation of the GLP-1 and NPY family of receptors could prove to be an important missing link between energy-related obesity and female reproductive dysfunction.

## Declaration of interest

The authors declare that there is no conflict of interest that could be perceived as prejudicing the impartiality of the work.

## Funding

These studies were supported by the Diabetes UK RD Lawrence Fellowship Grant to RCM and the Ulster University strategic funding.

## Author contribution statement

Dawood Khan helped in conceptualization, data curation, formal analysis, methodology, supervision, writing of the original draft, review and editing. Ananyaa Sridhar contributed to data curation, formal analysis, writing, review and editing. Charlotte R Moffett helped with conceptualization, methodology, funding acquisition, supervision, visualization, writing, review and editing.

## Data availability

The authors declare that the data supporting the findings of this study are available within the article. Any additional raw data supporting the conclusions of this article will be made available by the corresponding author, without undue reservation.
